# Supplementary material for: The Evolution and Characterization of the RNA Interference Pathways in Lophotrochozoa
Source: Genome Biol Evol. 2024 May 7;16(5):evae098. doi: 10.1093/gbe/evae098 (PMC11114477; doi:10.1093/gbe/evae098)
Supplement: evae098_Supplementary_Data [file evae098_supplementary_data.zip › Supplementary_materials.zip!/selected_miRNAs/NoGe_selected_miRNAs/mirdeep_pdf_files/NMRB01000426.1_Notospermus_geniculatus_strain_Ushimado_scaffold426,_whole_genome_shotgun_sequence_13050.pdf]

Provisional ID	:	NMRB01000426.1_Notospermus_geniculatus_strain_Ushimado_scaffold426,_whole_genome_shotgun_sequence_13050
Score total	:	154367.9
Score for star read(s)	:	3.9
Score for read counts	:	154360.4
Score for mfe	:	0.6
Score for randfold	:	0
Score for cons. seed	:	3
Total read count	:	302783
Mature read count	:	302744
Loop read count	:	3
Star read count	:	36

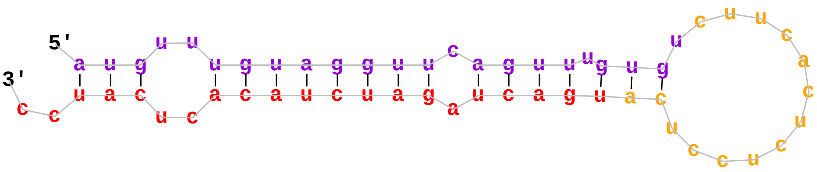

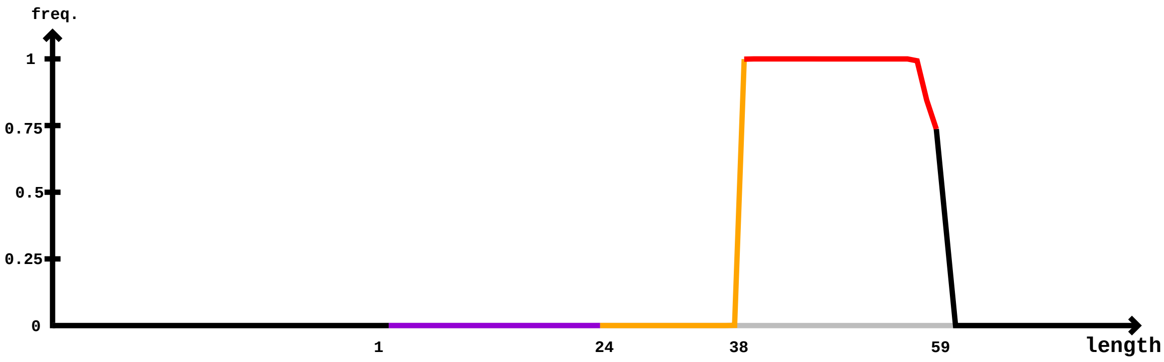

Star **Mature**

```
5'- agucagauuaugucaauaauccucaccacugaaauguuuguagguucaguuugugucuucacucuccucaugacuagaucuacacucaucccagugguugauggugaucua  -3'    obs
    agucagauuaugucaauaauccucaccacugaaauguuuguagguucaguuugugucuucacucuccucaugacuagaucuacacucaucccagugguugauggugaucua          exp
    ....(((((((.(((.........(((((((..(((..((((((((.((((.(((.............))))))).))))))))..)))..))))))))))..))))))).    reads      mm      sample
    .................................auguuuguagguucaguuugug........................................................    1          0       Mm3
    .......................................................ucuucacucuccucaugacuag..................................    2          0       Mm3
    .......................................................ucuucacucuccucaugacuaga.................................    5          0       Mm3
    .......................................................ucuucacucuccucaugacuagau................................    3          0       Mm3
    .......................................................ucuucacucuccucaugacuagaucuac............................    1          0       Mm3
    .....................................................................augacuagaucCacacucauc.....................    2          1       Mm3
    .....................................................................Cugacuagaucuacacucaucc....................    1          1       Mm3
    .....................................................................augacuagaucCacacucaucc....................    8          1       Mm3
    .....................................................................augacuagaucuacacucaucc....................    1          0       Mm3
    ......................................................................ugacuagaucuacacuca.......................    1          0       Mm3
    ......................................................................ugacuagaucCacacuca.......................    30         1       Mm3
    ......................................................................ugacuagaucuacacucau......................    1          0       Mm3
    ......................................................................ugacuagaucCacacucau......................    235        1       Mm3
    ......................................................................ugacuagaucuacacucauc.....................    51         0       Mm3
    ......................................................................ugacuagaucuacacucUuc.....................    1          1       Mm3
    ......................................................................ugacuagaucGacacucauc.....................    8          1       Mm3
    ......................................................................ugacuagaucCacacucauc.....................    3336       1       Mm3
    ......................................................................ugacuagaucuacacucUucc....................    1          1       Mm3
    ......................................................................uAacuagaucuacacucaucc....................    1          1       Mm3
    ......................................................................ugacuagaucuacacuUaucc....................    2          1       Mm3
    ......................................................................ugacuagaucCacacucaucc....................    31930      1       Mm3
    ......................................................................uUacuagaucuacacucaucc....................    1          1       Mm3
    ......................................................................ugacuagaucuacacucaucc....................    552        0       Mm3
    ......................................................................ugacuagaucuacacucaucU....................    13         1       Mm3
    ......................................................................ugacuagaucuacacucCucc....................    4          1       Mm3
    ......................................................................ugacuagUucuacacucaucc....................    1          1       Mm3
    ......................................................................ugacuagaucGacacucaucc....................    51         1       Mm3
    ......................................................................ugacuGgaucuacacucaucc....................    3          1       Mm3
    ......................................................................ugacuagaucuacacucGucc....................    2          1       Mm3
    ......................................................................ugacuagaucuacacucaucA....................    1          1       Mm3
    ......................................................................ugacuagaucuacacucauccU...................    184        1       Mm3
    ......................................................................ugacuagaucuacacucauccc...................    11         0       Mm3
    ......................................................................ugacuagaucuacacucauccA...................    12         1       Mm3
```

```
                                Star                                 Mature
agucagauuaugucaauaauccucaccacugaaauguuuguagguucaguuuguguuucacucucccucaugacuagaucuacacucaucccagugguugauggugaucua
.......................................................................ugacuagaucCacacucauccc...................   14       1   Mm3
.......................................................................ugacuagaucuacacucauccG...................   2        1   Mm3
.......................................................................ugacuagaucuacacucaucccU..................   1        1   Mm3
........................................................................gacuagaucCacacucau......................   1        1   Mm3
........................................................................gacuagaucCacacucauc.....................   6        1   Mm3
........................................................................gacuagaucCacacucaucc....................   35       1   Mm3
..........................................................................cuagaucCacacucaucc....................   1        1   Mm3

.................................auguuuguagguucaguuugu..........................................................   2        0   Fm3
.................................auguuuguagguucaguuugug.........................................................   2        0   Fm3
.................................auguuuguagguucaguuugugu........................................................   8        0   Fm3
.................................auguuuguagguucaguuuguguA.......................................................   1        1   Fm3
..................................uguuuguagguucaguuugugu........................................................   5        0   Fm3
.......................................................ucuucacucucccucaugacuagau................................   3        0   Fm3
.....................................................................caugacuagaucuacacucaucc....................   3        0   Fm3
......................................................................augacuagaucCacacucau......................   26       1   Fm3
......................................................................augacuagaucCacacucauc.....................   4        1   Fm3
......................................................................augacuagaucCacacucaucc....................   40       1   Fm3
......................................................................augacuagauAuacacucaucc....................   1        1   Fm3
......................................................................augacuagaucuacacucaucc....................   5        0   Fm3
......................................................................augacuagaucuacacucauccU...................   1        1   Fm3
.......................................................................ugacuagaucCacacuca.......................   583      1   Fm3
.......................................................................ugacuagaucuacacuca.......................   3        0   Fm3
.......................................................................ugacuagaucuacacucaA......................   1        1   Fm3
.......................................................................ugacuagaucCacacucau......................   38247    1   Fm3
.......................................................................ugacuagaucGacacucau......................   60       1   Fm3
.......................................................................ugacuagaucuacacucau......................   118      0   Fm3
.......................................................................ugacuagaucuacacuGau......................   1        1   Fm3
.......................................................................ugacuagaucuacacucUu......................   1        1   Fm3
.......................................................................ugacuagaucuacacuAauc.....................   2        1   Fm3
.......................................................................ugacuaAaucuacacucauc.....................   1        1   Fm3
.......................................................................ugacuagaucGacacucauc.....................   11       1   Fm3
.......................................................................ugacuagaucuacacucGuc.....................   1        1   Fm3
.......................................................................ugacuagaucuacacucauU.....................   1        1   Fm3
.......................................................................ugacuagaucuacacucaCc.....................   1        1   Fm3
.......................................................................ugacuagaucuacacucUuc.....................   1        1   Fm3
.......................................................................ugacuagaucCacacucauc.....................   7777     1   Fm3
.......................................................................ugacuagaucuacacucauc.....................   288      0   Fm3
.......................................................................ugacuGgaucuacacucauc.....................   1        1   Fm3
.......................................................................ugacuagaucuacacuAaucc....................   10       1   Fm3
.......................................................................uUacuagaucuacacucaucc....................   3        1   Fm3
.......................................................................ugacuagaucuacacucaCcc....................   1        1   Fm3
.......................................................................ugUcuagaucuacacucaucc....................   1        1   Fm3
.......................................................................ugacCagaucuacacucaucc....................   2        1   Fm3
.......................................................................uCacuagaucuacacucaucc....................   1        1   Fm3
.......................................................................ugacuagaucuacaGucaucc....................   1        1   Fm3
.......................................................................ugacuagCucuacacucaucc....................   1        1   Fm3
.......................................................................ugacuagaucuUcacucaucc....................   1        1   Fm3
.......................................................................ugacuaUaucuacacucaucc....................   1        1   Fm3
.......................................................................ugaAuagaucuacacucaucc....................   1        1   Fm3
.......................................................................ugacuagaucuacacucauGc....................   1        1   Fm3
.......................................................................ugacuCgaucuacacucaucc....................   1        1   Fm3
.......................................................................ugacuagaucCacacucaucc....................   109454   1   Fm3
.......................................................................ugacuGgaucuacacucaucc....................   5        1   Fm3
.......................................................................ugacuagGucuacacucaucc....................   1        1   Fm3
.......................................................................ugaGuagaucuacacucaucc....................   1        1   Fm3
.......................................................................ugCcuagaucuacacucaucc....................   1        1   Fm3
.......................................................................Ngacuagaucuacacucaucc....................   3        1   Fm3
.......................................................................ugacuagaucuacacCcaucc....................   1        1   Fm3
.......................................................................ugacuagaucuacacucaucU....................   51       1   Fm3
.......................................................................ugacuagaucuacacucCucc....................   1        1   Fm3
.......................................................................ugacuagauAuacacucaucc....................   2        1   Fm3
.......................................................................ugacuagaucuacacucaucc....................   2618     0   Fm3
.......................................................................ugacuagaucuacacucUucc....................   3        1   Fm3
.......................................................................ugacuagaucuacacuGaucc....................   2        1   Fm3
.......................................................................Cgacuagaucuacacucaucc....................   1        1   Fm3
.......................................................................ugacuagaucuacacucGucc....................   12       1   Fm3
.......................................................................ugacuagaucuacacuUaucc....................   6        1   Fm3
.......................................................................ugacuagaucuacacucauAc....................   1        1   Fm3
.......................................................................ugacuagaucGacacucaucc....................   187      1   Fm3
```

Star Mature

```
agucagauuaugucaauaauccucaccacugaaauguuuguagguucaguuuguguucuucacucuccucaugacuagaucuacacucaucccagugguugauggugaucua
......................................................................ugacuagaucuacacucauccU...................  884    1  Fm3
......................................................................ugacuagaucuacacucauccc...................  4      0  Fm3
......................................................................ugacuagaucuacacucauccA...................  56     1  Fm3
......................................................................ugacuagaucCacacucauccc...................  18     1  Fm3
......................................................................ugacuagaucuacacucaucccU..................  5      1  Fm3
.......................................................................gacuagaucCacacucau......................  38     1  Fm3
.......................................................................gacuagaucCacacucauc.....................  6      1  Fm3
.......................................................................gacuagaucuacacucaucc....................  2      0  Fm3
.......................................................................gacuagaucCacacucaucc....................  118    1  Fm3
.......................................................................gacuagaucuacacucauccU...................  2      1  Fm3
........................................................................acuagaucCacacucaucc....................  5      1  Fm3

.................................auguuuguagguucaguuugu.........................................................  2      0  Mm1
.....................................................................augacuagaucCacacucauc.....................  20     1  Mm1
.....................................................................augacuagaucuacacucaucc....................  16     0  Mm1
.....................................................................augacuagaucCacacucaucc....................  10     1  Mm1
......................................................................ugacuagaucGacacuca.......................  1      1  Mm1
......................................................................ugacuagaucCacacuca.......................  1309   1  Mm1
......................................................................ugacuagaucuacacuca.......................  1      0  Mm1
......................................................................ugacuagaucCacacucau......................  1692   1  Mm1
......................................................................ugacuagaucGacacucau......................  4      1  Mm1
......................................................................ugacuagaucuacacucau......................  9      0  Mm1
......................................................................ugacuagaucuacacucauU.....................  2      1  Mm1
......................................................................ugacuagaucCacacucauc.....................  13643  1  Mm1
......................................................................ugacuagaucGacacucauc.....................  22     1  Mm1
......................................................................ugacuagaucuacacucauc.....................  94     0  Mm1
......................................................................uAacuagaucuacacucaucc....................  1      1  Mm1
......................................................................ugacuagaucuacacucUucc....................  2      1  Mm1
......................................................................ugacuagaucuacacuUaucc....................  6      1  Mm1
......................................................................ugacuagaucuacacuAaucc....................  4      1  Mm1
......................................................................ugGcuagaucuacacucaucc....................  1      1  Mm1
......................................................................Ngacuagaucuacacucaucc....................  2      1  Mm1
......................................................................ugacuagaucuacacucaucc....................  2304   0  Mm1
......................................................................ugacuaNaucuacacucaucc....................  1      1  Mm1
......................................................................ugacuagaucuacacucGucc....................  10     1  Mm1
......................................................................ugacuagaucGacacucaucc....................  32     1  Mm1
......................................................................ugacuagaucCacacucaucc....................  26840  1  Mm1
......................................................................ugacuagaAcuacacucaucc....................  1      1  Mm1
......................................................................Agacuagaucuacacucaucc....................  2      1  Mm1
......................................................................ugacuagaucuacacuGaucc....................  3      1  Mm1
......................................................................ugacuagaucuacacucaucU....................  5      1  Mm1
......................................................................ugacuagaucuacacucCucc....................  7      1  Mm1
......................................................................ugUcuagaucuacacucaucc....................  2      1  Mm1
......................................................................ugacuagaucuacacucauUc....................  2      1  Mm1
......................................................................ugaAuagaucuacacucaucc....................  1      1  Mm1
......................................................................Cgacuagaucuacacucaucc....................  2      1  Mm1
......................................................................ugacuagaucuacacucauccU...................  158    1  Mm1
......................................................................ugacuagaucuacacucauccA...................  5      1  Mm1
.......................................................................gacuagaucCacacucauc.....................  14     1  Mm1
.......................................................................gacuagaucCacacucaucc....................  14     1  Mm1

................................aauguuuguagguucaguuugu.........................................................  1      0  Mm2
................................aauguuuguagguucaguuugug........................................................  2      0  Mm2
................................aauguuuguagguucaguuugugu.......................................................  1      0  Mm2
.................................auguuuguagguucaguuugugu.......................................................  5      0  Mm2
.......................................................ucuucacucuccucaugG......................................  2      1  Mm2
.......................................................ucuucacucuccucaugacu....................................  1      0  Mm2
.......................................................ucuucacucuccucaugacuag..................................  1      0  Mm2
.......................................................ucuucacucuccucaugacuaga.................................  5      0  Mm2
.......................................................ucuucacucuccucaugacuagau................................  2      0  Mm2
.......................................................ucuucacucuccucaugacuagauc...............................  1      0  Mm2
.......................................................ucuucacucuccucaugacuagaucuacacucaucU....................  1      1  Mm2
.......................................................ucuucacucuccucaugacuagaucuacacucauccU...................  1      1  Mm2
..............................................................ucuccucaugacuagaucuacacucaucc....................  1      0  Mm2
...............................................................cuccucaugacuagaucuacacucaucc....................  2      0  Mm2
...................................................................ucaugacuagaucuacacucaucc....................  1      0  Mm2
....................................................................caugacuagaucuacacucaucU....................  1      1  Mm2
....................................................................caugacuagaucuacacucaucc....................  1      0  Mm2
.....................................................................augacuagaucCacacucau......................  1      1  Mm2
.....................................................................augacuagaucuacacucaucc....................  2      0  Mm2
```

Star Mature

```
agucagauuaugucaauaauccucaccacugaaauguuuguagguucaguuuguguecuucacucuccucaugacuagaucuacacucaucccaguggu
```

```
agucagauuaugucaauaauccucaccacugaaauguuuguagguucaguuuguguucuucacucuccucaugacuagaucuacacucaucccagugguugauggugaucua
.....................................................................augacuagaucCacacucaucc....................  6      1   Mm2
......................................................................ugacuagaucCacacuca.......................  48     1   Mm2
......................................................................ugacuagaucuacacuca.......................  1      0   Mm2
......................................................................ugacuagaucCacacucau......................  2319   1   Mm2
......................................................................ugacuagaucGacacucau......................  4      1   Mm2
......................................................................ugacuagaucuacacucau......................  2      0   Mm2
......................................................................ugacuagaucGacacucauc.....................  3      1   Mm2
......................................................................ugacuagaucuacacucauc.....................  37     0   Mm2
......................................................................ugacuagaucCacacucauc.....................  765    1   Mm2
......................................................................ugacuagaucuacacucCucc....................  1      1   Mm2
......................................................................ugacuagaucuacacucGucc....................  4      1   Mm2
......................................................................ugGcuagaucuacacucaucc....................  1      1   Mm2
......................................................................ugacuagaucuGcacucaucc....................  1      1   Mm2
......................................................................ugacuagaucuacacucaucc....................  650    0   Mm2
......................................................................ugacuGgaucuacacucaucc....................  4      1   Mm2
......................................................................ugacuagaucuacacuUaucc....................  4      1   Mm2
......................................................................ugacuagaucGacacucaucc....................  28     1   Mm2
......................................................................ugacuagaucuacacucaucU....................  13     1   Mm2
......................................................................ugacuagaucCacacucaucc....................  12824  1   Mm2
......................................................................ugCcuagaucuacacucaucc....................  2      1   Mm2
......................................................................uCacuagaucuacacucaucc....................  1      1   Mm2
......................................................................ugacuagaucuacacuAaucc....................  1      1   Mm2
......................................................................ugacuagaucuacacuGaucc....................  1      1   Mm2
......................................................................ugacuagaucuacacucauccc...................  7      0   Mm2
......................................................................ugacuagaucuacacucauccU...................  66     1   Mm2
......................................................................ugacuagaucuacacucauccA...................  6      1   Mm2
......................................................................ugacuagaucuacacucauccG...................  3      1   Mm2
......................................................................ugacuagaucCacacucauccc...................  20     1   Mm2
......................................................................ugacuagaucuacacucaucccU..................  2      1   Mm2
......................................................................ugacuagaucuacacucauccAa..................  3      1   Mm2
.......................................................................gacuagaucCacacucaucc....................  9      1   Mm2

.................................auguuuguagguucaguuugu.........................................................  3      0   Fm1
.................................auguuuguagguucaguuugug........................................................  3      0   Fm1
.......................................................ucuucacucuccucaugacua...................................  1      0   Fm1
.....................................................................augacuagaucuacacucauc.....................  8      0   Fm1
.....................................................................augacuagaucCacacucauc.....................  10     1   Fm1
.....................................................................augacuagaucCacacucaucc....................  3      1   Fm1
.....................................................................augacuagaucuacacucaucc....................  2      0   Fm1
......................................................................ugacuagaucGacacuca.......................  1      1   Fm1
......................................................................ugacuagaucCacacuca.......................  104    1   Fm1
......................................................................ugacuagaucGacacucau......................  3      1   Fm1
......................................................................ugacuagaucuacacucau......................  13     0   Fm1
......................................................................ugacuagaucCacacucau......................  1663   1   Fm1
......................................................................ugacuagaucCacacucauc.....................  2197   1   Fm1
......................................................................ugacuagaucuacacucauU.....................  1      1   Fm1
......................................................................ugacuagaucuacacucGuc.....................  2      1   Fm1
......................................................................ugacuagaucuacacucUuc.....................  1      1   Fm1
......................................................................ugUcuagaucuacacucauc.....................  1      1   Fm1
......................................................................Ggacuagaucuacacucauc.....................  1      1   Fm1
......................................................................ugacuagaucuacacucauc.....................  182    0   Fm1
......................................................................ugacuagaucGacacucauc.....................  2      1   Fm1
......................................................................ugacuaAaucuacacucauc.....................  1      1   Fm1
......................................................................ugacuagaucuacacGcauc.....................  1      1   Fm1
......................................................................ugacuagUucuacacucaucc....................  1      1   Fm1
......................................................................ugacuagaucuacacuGaucc....................  1      1   Fm1
......................................................................ugacuagaucuacacucaucU....................  6      1   Fm1
......................................................................ugacuagaucuacacuAaucc....................  5      1   Fm1
......................................................................ugacuagaucCacacucaucc....................  13070  1   Fm1
......................................................................ugacuagaucuacacucCucc....................  1      1   Fm1
......................................................................ugacCagaucuacacucaucc....................  1      1   Fm1
......................................................................ugacuaUaucuacacucaucc....................  2      1   Fm1
......................................................................uAacuagaucuacacucaucc....................  1      1   Fm1
......................................................................ugacuagaucGacacucaucc....................  21     1   Fm1
......................................................................Ngacuagaucuacacucaucc....................  1      1   Fm1
......................................................................ugacuagaucuacacucaucc....................  1511   0   Fm1
......................................................................ugacuagaucuacacuUaucc....................  4      1   Fm1
......................................................................ugacuagaucuacacucGucc....................  7      1   Fm1
......................................................................ugacuagaucuacacucauGc....................  1      1   Fm1
......................................................................ugCcuagaucuacacucaucc....................  1      1   Fm1
```

Star Mature

```
agucagauuaugucaauaauccucaccacugaaauguuuguagguucaguuuguguc uucacucucccucaugacuagaucuacacucauccaguggu ugauggugaucua
```

```
agucagauuaugucaauaauccucaccacugaaauguuuguagguucaguuugugucuucacucucccucaugacuagaucuacacucaucccagugguugauggugaucua
.......................................................................ugaAuagaucuacacucaucc....................    1      1     Fm1
.......................................................................ugUcuagaucuacacucaucc....................    1      1     Fm1
.......................................................................uUacuagaucuacacucaucc....................    1      1     Fm1
.......................................................................ugacuagaucuacacucUucc....................    2      1     Fm1
.......................................................................ugacuagaucCacacucauccc...................    6      1     Fm1
.......................................................................ugacuagaucuacacucauccG...................    7      1     Fm1
.......................................................................ugacuagaucuacacucauccU...................    174    1     Fm1
.......................................................................ugacuagaucuacacucauccc...................    4      0     Fm1
.......................................................................ugacuagaucuacacucauccA...................    14     1     Fm1
........................................................................gacuagaucCacacucauc.....................    1      1     Fm1
........................................................................gacuagaucCacacucaucc....................    5      1     Fm1

......................................................................augacuagaucuacacucaucc....................    15     0     Fm2
.......................................................................ugacuagaucCacacuca.......................    30     1     Fm2
.......................................................................ugacuagaucCacacucau......................    312    1     Fm2
.......................................................................ugacuagaucuacacucau......................    1      0     Fm2
.......................................................................ugacuagaucuacacuUauc.....................    1      1     Fm2
.......................................................................ugacuagaucuacacucauc.....................    27     0     Fm2
.......................................................................ugacuagaucGacacucauc.....................    12     1     Fm2
.......................................................................ugacuagaucCacacucauc.....................    4299   1     Fm2
.......................................................................ugacuagaucuaUacucaucc....................    1      1     Fm2
.......................................................................ugacuagaucGacacucaucc....................    21     1     Fm2
.......................................................................ugacuagaucuacacucCucc....................    1      1     Fm2
.......................................................................ugacuagaucCacacucaucc....................    17744  1     Fm2
.......................................................................ugacuagaucuacacucaCcc....................    1      1     Fm2
.......................................................................ugacuNgaucuacacucaucc....................    1      1     Fm2
.......................................................................ugacuagaucuacacuUaucc....................    1      1     Fm2
.......................................................................ugacuagaucuacacucaucc....................    807    0     Fm2
.......................................................................ugacuagaucuacacuAaucc....................    3      1     Fm2
.......................................................................Ngacuagaucuacacucaucc....................    1      1     Fm2
.......................................................................ugacuagaucuacacucGucc....................    2      1     Fm2
.......................................................................ugacuagaucuacacucauUc....................    1      1     Fm2
.......................................................................ugacuagaucuacacucauccU...................    109    1     Fm2
.......................................................................ugacuagaucuacacucauccG...................    8      1     Fm2
.......................................................................ugacuagaucuacacucauccA...................    5      1     Fm2
.......................................................................ugacuagaucCacacucauccc...................    12     1     Fm2
........................................................................gacuagaucCacacucaucc....................    4      1     Fm2
.........................................................................acuagaucCacacucauc.....................    1      1     Fm2
```
